# Antimicrobial Efficacy of Endogenous Blue Light Photoinactivation (400–470 nm) Against *Escherichia coli*: A Systematic Review of In Vitro Evidence and Clinical Implications

**DOI:** 10.3390/medsci14020261

**Published:** 2026-05-20

**Authors:** Diego Antônio C. P. Gomes Mello, João Pedro R. Afonso, Everton Edgar Carvalho, Hustênio Abílio Appelt Filho, Jairo Belém Soares Ribeiro Júnior, Larissa Rodrigues Alves, Mickael Breno Godoi Sousa, Salomão Antonio Oliveira, Guilherme Quireza Silva, Rafael Souza Bueno, Tiago Vieira Fernandes, Daniel Grossi Marconi, Rodrigo Antônio C. Andraus, Carlos Hassel Mendes Silva, Deise A. A. Pires Oliveira, Iransé Oliveira-Silva, Rodrigo Franco Oliveira, Orlando Aguirre Guedes, Wilson Rodrigues Freitas Júnior, Juan Jose Uriarte, Luis V. F. Oliveira, Luis Gustavo Morato Toledo

**Affiliations:** 1 Health Sciences Graduate Program (PPGCS), Faculty of Medical Sciences of Santa Casa de São Paulo, São Paulo 01225-010, SP, Brazil; diego0611escs@hotmail.com (D.A.C.P.G.M.); hustenio@hotmail.com (H.A.A.F.); drbelembelem@hotmail.com (J.B.S.R.J.); lari.ralves@gmail.com (L.R.A.); salomaoantoniodeoliveira@gmail.com (S.A.O.); guilherme.quireza@gmail.com (G.Q.S.); bueno.rafaeldesouza@gmail.com (R.S.B.); tiago.vieirafernandes@hotmail.com (T.V.F.); carloshmendes@unievangelica.edu.br (C.H.M.S.); wilson.freitas@fcmsantacasasp.edu.br (W.R.F.J.); luis.toledo@fcmsantacasasp.edu.br (L.G.M.T.); 2 Human Movement and Rehabilitation Graduate Program (PPGMHR), Evangelical University of Goiás (UniEVANGELICA), Anápolis 75083-515, GO, Brazil; joaopedro180599@gmail.com (J.P.R.A.); edgardecarvalho@fampfaculdade.com.br (E.E.C.); mckb.inf@gmail.com (M.B.G.S.); rodrigoandraus@gmail.com (R.A.C.A.); deisepyres@gmail.com (D.A.A.P.O.); iranseoliveira@hotmail.com (I.O.-S.); rodrigofranco65@gmail.com (R.F.O.); 3 Hospital de Câncer de Barretos—Hospital do Amor, Barretos 14784-400, SP, Brazil; dgmarconi@gmail.com; 4 Dentistry Graduate Program (PPGO), Evangelical University of Goiás (UniEVANGELICA), Anápolis 01225-010, GO, Brazil; orlandoaguedes@gmail.com; 5 ARIES Research Group, Escuela Politécnica Superior, Universidad Nebrija, 28015 Madrid, Spain; uriarte.juanjo@gmail.com

**Keywords:** *Escherichia coli*, antimicrobial blue light, endogenous photoinactivation, 405 nm, reactive oxygen species, multidrug resistance

## Abstract

Background/Objectives: The increased prevalence of multidrug-resistant *Escherichia coli* and carbapenemase-producing Enterobacteriaceae poses a critical threat to global health and food safety. Antimicrobial Blue Light (aBL) in the 400–470 nm spectrum has emerged as a promising, chemical-free disinfection strategy that targets intracellular porphyrins and flavins to induce oxidative stress. However, the influence of wavelength, dosimetry, and environmental stressors on endogenous photoinactivation remains poorly standardized regarding optical parameters and biological exposure protocols. This systematic review aimed to evaluate the antimicrobial efficacy of pure blue light (400–470 nm) against *E. coli* across various phenotypes and environmental conditions, excluding the use of exogenous photosensitizers. Methods: PubMed, Scopus, and Web of Science were searched for studies that utilized 400–470 nm light as an antimicrobial agent against *E. coli*. Data extraction focused on spectral efficiency, total fluence (J/cm^2^), and log_10_ reduction. The Risk of Bias was assessed using an adapted Office of Health Assessment and Translation tool for in vitro studies. Results: Synthesis of 11 high-quality studies indicated that wavelengths near 405 nm have the highest germicidal efficiency due to the Soret band absorption of endogenous porphyrins. Efficacy is highly dose-dependent: significant log_10_ reductions were achieved in planktonic cells, although biofilms required substantially higher fluences. Sub-lethal environmental stressors such as acidic pH, high salinity, and thermal fluctuations demonstrated a synergistic effect, which significantly enhanced the rate of photoinactivation. Multidrug-resistant and carbapenemase-producing Enterobacteriaceae strains showed similar susceptibility to aBL relative to antibiotic-sensitive strains, suggesting no cross-resistance between light and traditional drugs. Conclusions: Endogenous blue light is a highly effective, non-thermal technology for *E. coli* decontamination. Its efficacy is modulated by the interplay between optical parameters and environmental conditions. These findings provide a framework for the development of standardized protocols for applying aBL to clinical wound care and food industry use cases. They also highlight the potential of aBL as a critical tool in the post-antibiotic era. This systematic review was registered in the International prospective register of systematic reviews (PROSPERO) under protocol CRD420261331871.

## 1. Introduction

The global increase in the prevalence of antimicrobial resistance represents one of the most critical threats to public health and food security in the 21st century [1,2]. Recent studies have shown that the inability to treat common bacterial infections—due to microorganism resistance against conventional antibiotics—may result in a regression to the pre-antibiotic era, where routine medical procedures would become high-risk [3,4,5].

*Escherichia coli* is a unique bacterial species that plays a central role in epidemiological surveillance. It is a highly disseminated opportunistic pathogen and the primary etiological agent of urinary tract infections, septicemia, and foodborne outbreaks. It also has a genetic variation that allows it to transition between commensalism and acute virulence with extreme efficacy [6,7,8].

The dissemination of high-risk clones and strains producing carbapenemases and extended-spectrum beta-lactamases has worsened the problem. These superbugs not only develop resistance against last-line antibiotics but also have a remarkable capacity for biofilm formation [9,10]. The biofilm, a self-produced extracellular polymeric matrix, confers a phenotypic tolerance to *E. coli* that can be up to 1000 times higher than that observed in a planktonic state to protect the microorganisms against the immune system and chemical disinfectants of the host [11,12]. This structural resistance makes the control of *E. coli* a persistent challenge in hospital environments (through healthcare-associated infections) and in the food processing industry [13,14].

Given the urgent need for alternative therapies that do not rely on traditional pharmacological pathways, antimicrobial aBL, particularly in the 400–470 nm spectrum, emerges as a promising and technologically sustainable alternative [15,16,17]. Unlike antimicrobial photodynamic therapy, which requires the administration of external dyes (photosensitizers), aBL acts on endogenous chromophores present within the bacterial cell [17,18].

The fundamental mechanism involves the photoexcitation of intracellular porphyrins and flavins, which trigger a cascade of reactions that generate reactive oxygen species after absorbing photons [19]. This oxidative stress promotes multifocal damage, including lipid peroxidation and rupture of the cytoplasmic membrane. Cell death ultimately ensues independent of the enzymatic resistance or efflux mechanisms of the bacteria [20].

Despite growing evidence regarding the bactericidal potential of aBL, the experimental results available in the scientific literature are significantly variable. Inactivation efficacy is directly influenced by a multiplicity of factors, such as the specific wavelength used, power density, total energy dose administered, and the complexity of the matrix in which the bacteria are embedded [21,22,23,24]. Consequently, systematic consolidation of the current findings is necessary to discern the limits and potential of this technology against *Escherichia coli*.

The objective of this systematic review was to critically evaluate the efficacy of aBL irradiation in inactivating *Escherichia coli* by analyzing how different physical parameters and environmental conditions, ranging from planktonic states and biofilms to complex matrices such as food and biological tissues, modulate the bactericidal response. Through this work, we intend to establish evidence to guide future clinical and industrial applications of this promising, non-invasive therapy. While general reviews on Antimicrobial Blue Light (aBL) exist, this systematic review provides a distinct contribution by strictly isolating the pure endogenous photoinactivation mechanisms against E. coli, intentionally excluding any exogenous photosensitizers (aPDT) or chemical adjuvants to map the bacterium’s baseline susceptibility. Furthermore, we synthesize the highly heterogeneous data into an unprecedented, structured dosimetric framework, providing clear, actionable optical thresholds categorized by biological target states (planktonic vs. biofilms) and complex matrices.

## 2. Materials and Methods

### 2.1. Study Design and Protocol Registration

This systematic review was conducted following the Preferred Reporting Items for Systematic Reviews and Meta-Analyses (2020) guidelines [25]. The protocol was prospectively registered in the International Prospective Register of Systematic Reviews (PROSPERO) under the identification number CRD420261331871.

### 2.2. Search Strategy and Information Sources

A systematic electronic search was performed in PubMed/MEDLINE, Scopus, and Web of Science for studies published up to March 2026. To ensure full reproducibility, the complete Boolean search syntax applied across the databases was structured as follows: For PubMed/MEDLINE, the exact string used was (“*Escherichia coli*” [Mesh] OR “*Escherichia coli*”) AND (“blue light” OR “405 nm” OR “photoinactivation”) AND (“endogenous”). For Web of Science and Scopus, the syntax applied was TS = (“*Escherichia coli*” AND (“blue light” OR “405 nm” OR “photoinactivation”) AND “endogenous”). No automation filters regarding publication date or article type were applied during the primary electronic search to avoid the accidental omission of relevant bench studies. Gray literature sources, including Google Scholar and specialized repositories, were manually screened for additional relevant records. Although the initial database query did not restrict language metadata, the final eligibility assessment focused on peer-reviewed articles published in English to guarantee standardized reporting of physical, optical, and dosimetric parameters. Additionally, a manual search of the reference lists of all included studies was performed to identify any further eligible literature.

### 2.3. Eligibility Criteria (PICO)

Studies were included based on the following criteria: Population (P): Any strain of *Escherichia coli* (standard, clinical isolates, or multidrug-resistant/carbapenemase-producing Enterobacteriaceae); Intervention (I): Application of aBL (400–470 nm) as the sole antimicrobial agent; Comparator (C): Non-irradiated dark controls or comparisons between different light parameters; Outcome (O): Quantitative bacterial reduction (CFU/mL or CFU/cm^2^) expressed as log10 reduction. Regarding the exclusion criteria, studies involving exogenous photosensitizers (photodynamic therapy), photocatalysts, or chemical adjuvants were excluded to strictly isolate the endogenous effects of the light. Furthermore, secondary literature (such as systematic reviews, narrative reviews, and meta-analyses), conference abstracts, proceeding papers, and short communications were excluded to ensure that only primary, fully detailed experimental data were synthesized. The eligibility was restricted to peer-reviewed articles published in English; this language boundary was maintained to avoid methodological bias arising from the potential misinterpretation of highly specific physical, mathematical, and optical dosimetric parameters reported in other languages, ensuring standardized data extraction.

### 2.4. Study Selection and Data Extraction

Two reviewers independently performed title and abstract screening, followed by full-text review. Any disagreements were resolved through consensus or by a third reviewer. Data were extracted into a standardized spreadsheet. They included the following: (a) author/year; (b) bacterial strain and phase; (c) light parameters (wavelength, irradiance, and fluence); and (d) main bactericidal results.

### 2.5. Quality and Risk of Bias Assessment

The methodological quality of the included studies was appraised using an adapted version of the Office of Health Assessment and Translation Risk of Bias Tool for in vitro studies [26]. The assessment focused on internal validity domains, such as light dosimetry precision, temperature control during irradiation, and standardized microbial quantification methods. The internal validity of the included studies was evaluated using the Office of Health Assessment and Translation (OHAT) Risk of Bias Tool for in vitro studies, with each article independently assessed by two reviewers across specific domains.

These domains encompassed Selection Bias (randomization and allocation concealment), Performance Bias (blinding of research personnel and consistency of experimental conditions), Detection Bias (blinding of outcome assessors and validity of outcome assessment methods), and Attrition/Exclusion Bias (completeness of data reporting). Each domain was categorized into one of four levels, ‘Definitely Low Risk,’ ‘Probably Low Risk,’ ‘Probably High Risk,’ or ‘Definitely High Risk’, and subsequently integrated to determine the overall evidence quality. Following the OHAT framework, studies were classified into Tiers (1, 2, or 3) based on the frequency of ‘Low Risk’ ratings in key domains, with Exposure Confidence (LED calibration) and Outcome Confidence (CFU counting) pre-defined as the most critical factors for ensuring the validity of the observed photodynamic effects.

For the study encompassing both in vitro and in vivo methodologies [26], the quality assessment was conducted separately for each experimental arm. While the laboratory components were evaluated using the adapted OHAT tool for in vitro studies, the animal model segments were assessed based on specific bias domains for in vivo research, such as randomization and allocation concealment, ensuring a robust evaluation across different study designs.

### 2.6. Data Synthesis

A narrative synthesis was performed to summarize and compare the findings across the included primary studies. Data were systematically categorized and discussed based on wavelength efficiency (e.g., 405 nm vs. 450 nm), the relationship between energy dose (fluence) and bacterial kill rate, and the influence of sub-lethal environmental stressors (pH, temperature, and matrix complexity). A formal quantitative synthesis (meta-analysis) was determined to be mathematically unfeasible and conceptually inappropriate due to critical methodological, biological, and dosimetric heterogeneity among the experiments. The optical parameters varied drastically, with irradiances ranging from 2.1 to 155 mW/cm^2^ and total fluences spanning from 2.3 to 540 J/cm^2^. Furthermore, the primary studies utilized highly diverse bacterial strains evaluated across distinct organizational or metabolic states (including active logarithmic phase cells, tolerant stationary-phase cells, and 72 h mature biofilms). Methodological variation was also present in the experimental models, which involved deeply contrasting biological and food matrices (such as simplified agar surfaces, liquid PBS, whole blood, beef, and cow’s milk), as well as disparate outcome reporting units (ranging from absolute log10 reduction in CFU/mL to percentage viability or biomass seeding inhibition). Pooling these fundamentally incompatible datasets into a single statistical model would yield extreme, uninterpretable statistical heterogeneity (I^2^) and misleading pooled effect sizes, thereby fully justifying a strictly narrative synthesis approach.

## 3. Results

### 3.1. Study Selection Flow

The systematic search was conducted across three main databases: PubMed, Web of Science, and Scopus. Initially, 691 articles were identified, with 289 from Web of Science, 111 from PubMed, and 291 from Scopus. After removing 217 duplicates, 474 titles and abstracts remained for initial screening.

**Figure 1:**
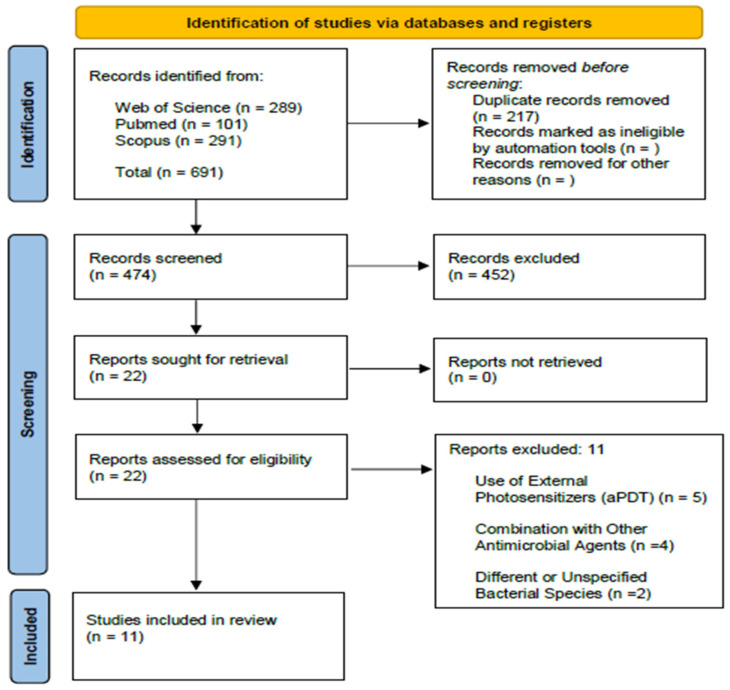
Flow diagram of the current systematic review conducted according to the Preferred Reporting Items for Systematic Reviews and Meta-analysis (PRISMA) guidelines.

After applying the inclusion and exclusion criteria based on reading titles and abstracts, 22 articles were selected for full-text review. At the end of the critical analysis and eligibility process, 11 articles met all requirements and were included in the final qualitative synthesis of this review (Figure 1).

### 3.2. Characteristics of the Included Studies

The 11 selected studies were published between 2013 and 2022 and were experimental (in vitro and in vivo). Table 1 presents a summary of the extracted data, detailing the *Escherichia coli* strains used, lighting parameters, and main biological outcomes.

### 3.3. Analysis of the Risk of Bias

The assessment was conducted in accordance with the Office of Health Assessment and Translation (OHAT) framework and demonstrated high methodological consistency across the 11 selected studies, with the majority classified as “Tier 2” (moderate-to-high confidence). All included studies exhibited a low Risk of Bias in critical domains for internal validity, specifically regarding Exposure Confidence and Outcome Confidence. This reliability stems from the use of calibrated LED systems and gold-standard microbiological methods, such as Colony Forming Unit (CFU) counts (Table 2).

Furthermore, recent studies reported strategies to mitigate thermal bias—including cooling systems and continuous temperature monitoring—thereby reinforcing the precision of the observed photodynamic effects. However, a lack of detailed reporting regarding randomization and blinding (both Performance and Detection Bias) was a common limitation identified across nearly all analyzed in vitro experiments. Despite these reporting gaps, the robustness of the outcome data and the absence of selective reporting provide a solid foundation for the evidence presented in this review.

### 3.4. Analysis of Irradiation Parameters

The evaluated wavelengths were mostly concentrated in the 405 nm range (seven studies), with variations between 400 and 470 nm in specific studies. The applied irradiance across the included experiments varied widely, ranging from 2.1 to 155 mW/cm^2^, with a calculated median of approximately 60 mW/cm^2^. Similarly, total energy doses (fluence) exhibited a broad distribution, ranging from low-level exposures of 2.3 J/cm^2^ to robust exposures of 540 J/cm^2^. This dosimetric analysis demonstrates that the inactivation of *E. coli* was directly proportional to the total photon dose delivered to the system, although the rate of inactivation was modulated by the specific power density used.

### 3.5. Susceptibility in Different Bacterial States

The results indicate that *E. coli* has different resistance profiles based on its organizational state. Significant reductions (between 1.4 and 5.3 log_10_) were observed in the planktonic state, especially during the active growth phase (logarithmic). For the biofilm form, studies focused on 72 h organizations demonstrated that aBL is effective not only in reducing the internal viability of the matrix but mainly in inhibiting bacterial dispersal (seeding), with reductions exceeding 90% [27,37].

### 3.6. Impact of Matrices and Stress Factors

The effectiveness of light was significantly influenced by the exposure medium. In transparent or simplified media (phosphate-buffered saline/agar), inactivation was maximal. In complex matrices, such as beef, whole milk, and blood, effectiveness was reduced due to the absorption and scattering of light by proteins, fats, and hemoglobin. In contrast, the combination of aBL with thermal stress (45 °C), saline, or acidic stress resulted in a synergistic effect that accelerated cell death [32].

### 3.7. Resistance and Security

No development of resistance to aBL was observed in *E. coli* after multiple successive exposures [33]. Furthermore, in vivo application to wound models demonstrated that the bactericidal doses were selective and caused no significant collateral damage to host tissues.

## 4. Discussion

### 4.1. Biophysics of Photoinactivation and the Soret Band

The effectiveness of aBL on *E. coli* is not a random event but rather a direct consequence of the metabolic organization of the bacterium. Unlike traditional photodynamic therapy, aBL takes advantage of endogenous photosensitizers such as protoporphyrin IX and coproporphyrin, which naturally reside in the cytoplasm [28,35]. By comparing different spectra, it has been shown that only the blue range possesses the photonic energy necessary to trigger the electronic transition of these chromophores [34]. This phenomenon occurs because of the Soret band, a critical electromagnetic absorption peak observed around 405 nm, which is the most effective parameter [28,32].

These porphyrins transition to a triplet excitation state when they absorb photons at this frequency and, in turn, react with molecular oxygen to generate reactive oxygen species. The impact of this process is aggressive for the microorganism, as singlet oxygen and hydroxyl radicals initiate a lipid peroxidation chain reaction [28,29,36]. This chemical reaction not only compromises the structural integrity of the cytoplasmic membrane but also inactivates essential transport proteins and respiratory enzymes [29]. By acting through a multifocal attack on basal metabolism and the physical barrier of the cell, aBL is effective at preventing *E. coli* from overcoming the obstacles through mutations in protein receptors that are the target of conventional antibiotics. The photochemical aggression affects the main components of bacterial viability [33,37].

### 4.2. Effectiveness Against High-Risk Clones and Bacterial Resistance

The clinical relevance of aBL is amplified by its ability to inactivate *E. coli* strains carrying important resistance genes. In the presence of carbapenemases (NDM, OXA-48) or resistance to colistin (mcr-1), no protection against phototherapy was observed [28,37]. This suggests that genetic mutations altering antibiotic binding sites do not interfere with porphyrin accumulation or sensitivity to oxidative stress.

Additionally, the rapid development of resistance is one of the greatest challenges in modern antibiotic therapy. However, *E. coli* does not develop functional resistance to aBL even after 15 successive irradiation cycles [33]. This is attributed to the multifocal impact of light on the bacterial cell, making it unlikely that a single mutation would confer protection against widespread oxidative damage.

Considering the diversity of *Escherichia coli*, it is important to note that this species encompasses a wide range of pathotypes, such as enterohemorrhagic (EHEC), enterotoxigenic (ETEC), enteropathogenic (EPEC), and uropathogenic (UPEC) strains.

Although the included studies focused predominantly on UPEC, EAEC, and specific multidrug-resistant (MDR) clones (e.g., ST131 and ST648), the fundamental mechanism of aBL the photoexcitation of endogenous porphyrins is a metabolic feature widely conserved across the species. Therefore, the bactericidal efficacy observed in these studies likely extends to other virulent strains, such as *E. coli* O157:H7, given that the intracellular accumulation of chromophores and the subsequent generation of reactive oxygen species (ROS) are not pathotype-specific, but rather related to basal bacterial physiology and the availability of toxicity.

### 4.3. Influence of Metabolic and Organizational State

The transition of *E. coli* from a planktonic state to an organized mature biofilm introduces complexities that extend beyond mere mechanical resistance. The extracellular polymeric substance matrix acts as an optical filter, promoting the scattering and attenuation of photons before they reach the basal layers of the community [27,37]. They do not completely eradicate biomass, but photons interrupt the biofilm life cycle by inhibiting bacterial dispersal (seeding) [37]. The 405 nm aBL may deactivate cell detachment mechanisms and prevent localized colonization from evolving into systemic bacteremia, even if a residual biofilm remains attached to the surface [27,37].

Regarding biological analysis, the effectiveness of aBL is intrinsically modulated by the metabolic gradient present in the biofilm. Light susceptibility depends on the replicative state. Cells in the logarithmic phase have accelerated oxidative metabolism and are critically vulnerable, whereas those in the stationary phase show marked tolerance [30]. This difference is attributed to the downregulation of endogenous porphyrin synthesis during states of low metabolic activity, which limits the availability of intracellular photosensitizers. Furthermore, the activation of stress response regulators, such as the sigma factor RpoS, provides protection against oxidative damage, positively regulating antioxidant enzymes (e.g., catalase and superoxide dismutase) and increasing the efficiency of DNA repair systems. Therefore, biofilm maturity creates a set of persistent or dormant cells that exhibit slow metabolism, in addition to robust enzymatic defenses, which consequently presents fewer active molecular targets for photoexcitation [22,29,30].

Due to these variations, the clinical success of the therapy depends on a rigorous calibration of fluence (J/cm^2^), wherein lower doses can inhibit surface scattering, but only high and possibly fractionated doses can overcome the repair threshold of stationary basal cells to ensure complete sterilization of the matrix [31,33].

To establish a reliable clinical and industrial guide, the methodological foundation of this dosimetric framework was strictly derived by synthesizing empirical data exclusively from the high-rigor primary studies categorized as Tier 1 or Tier 2 in the OHAT Risk of Bias assessment. By correlating the lowest fluences that successfully induced significant bacterial clearance (≥3log10 reduction or >90% biofilm seeding inhibition) across these highly controlled experimental designs, we mapped the overlapping operational windows presented in Table 3.

The interplay between irradiance (mW/cm^2^) and total fluence (J/cm^2^) is a critical determinant of therapeutic success. While fluence represents the total photon budget delivered, high irradiance is necessary to overcome the optical shielding of the extracellular matrix and the rapid DNA repair mechanisms of stationary-phase cells. As synthesized in Table 3, clinical protocols should prioritize high irradiance to ensure that the rate of oxidative damage exceeds the bacterial antioxidant response.

### 4.4. Impact of Biological Matrices and the Attenuation Phenomenon

The practical application of aBL has optical challenges when transposed from laboratory settings to real matrices. The results for beef, milk, and blood reveal that aBL undergoes intense scattering and competitive absorption [29,31,35]. In these contexts, chromophores such as hemoglobin or fat globules compete with bacterial porphyrins for photon absorption. This shows that the characteristics of the contaminated surface must also be evaluated.

The Hurdle Technology strategy shows promise for mitigating this limitation [32]. By combining light with thermal stress (45 °C) or acidification, the required energy dose can be reduced. This makes the process more efficient, even in less transparent media [32]. However, aBL may not be used as an isolated solution but as part of a multimodal decontamination protocol to ensure greater decontamination effectiveness.

### 4.5. Clinical Perspectives and Cellular Safety

Therefore, the safety of aBL for mammalian tissues is important for its clinical application. Effective and safe therapeutic windows require doses that inactivate *E. coli* (such as 45–60 J/cm^2^) to be below the toxicity threshold for fibroblasts and keratinocytes [33,36]. The demonstration of efficacy in cutaneous wound models validates the technology for the treatment of skin and soft tissue infections. The use of diffusing optical fibers opens the way for interventions for internal cavities, such as the urinary tract, which is frequently colonized by pathogenic *E. coli* [31].

However, translating these findings directly to human clinical settings requires extreme caution. Since the vast majority of the current evidence is derived from in vitro models, key physiological factors such as in-tissue light attenuation, optical scattering, and competitive photon absorption by mammalian chromophores (e.g., hemoglobin and melanin) remain unresolved challenges. Therefore, the dosimetric parameters proposed here serve as a preliminary baseline framework that requires extensive future clinical validation in vivo.

To better contextualize the clinical relevance of our findings, it is essential to correlate the *E. coli* pathotypes identified in the primary studies with their respective clinical manifestations. The evidence synthesized in this review encompasses uropathogenic *E. coli* (UPEC), which is the primary etiologic agent of urinary tract infections (UTIs); enteroaggregative *E. coli* (EAEC), associated with persistent diarrhea; and several multidrug-resistant (MDR) lineages, such as the ST131 and ST648 clones, which are frequently implicated in surgical site infections, bloodstream infections, and hospital-acquired pneumonia, according Table 4. The high susceptibility of these diverse pathotypes to aBL fluences suggests that this technology could be tailored for specific clinical scenarios, such as the decontamination of chronic wounds, the treatment of localized superficial infections, or the environmental disinfection of clinical surfaces frequently contaminated with enteric pathogens.

### 4.6. Potential of aBL Against Other Pathogenic Bacteria and Future Perspectives

Although this systematic review focused on the efficacy of aBL against *E. coli*, the photochemical principles of endogenous photoinactivation suggest a broader antimicrobial potential. The excitation of intracellular porphyrins (such as protoporphyrin IX and coproporphyrin) and flavins is a mechanism shared by several other pathogens of clinical and food safety relevance [38,39]. Recent literature indicates that aBL is effective against a wide spectrum of bacteria, including Gram-positive species such as *Staphylococcus aureus* (including MRSA) and *Enterococcus faecalis*, as well as other Gram-negative ESKAPE pathogens, such as *Pseudomonas aeruginosa* and *Acinetobacter baumannii* [40,41,42].

The multifocal nature of the oxidative stress induced by aBL targeting lipids, proteins, and DNA simultaneously minimizes the likelihood of bacteria developing functional resistance. This is particularly relevant as photoinactivation does not rely on specific enzymatic targets susceptible to mutation, a finding consistent with the lack of resistance observed in the *E. coli* studies analyzed here. Furthermore, the integration of aBL into hurdle technology frameworks combining light with thermal or acidic stress shows promise in enhancing bacterial kill rates while reducing the required energy dose [32,43,44]. Therefore, the dosimetric framework established for *E. coli* serves as a critical proof-of-concept for the development of multi-target decontamination protocols. Future research should prioritize well-designed clinical trials to validate these theoretical parameters in human tissue models and explore the synergistic effects of aBL with other non-pharmacological therapies to combat the global threat of multidrug resistance.

## 5. Conclusions

This systematic review suggests that Antimicrobial Blue Light (aBL) represents a promising and innovative approach for the inactivation of *E. coli*, including multidrug-resistant and biofilm-producing strains. The mechanism, based on the photoexcitation of endogenous compounds and subsequent cellular oxidation, appears to operate independently of traditional antibiotic resistance mechanisms. However, the data synthesized here are derived predominantly from in vitro studies, and the antimicrobial performance is highly sensitive to variations in optical dosimetry and the optical density of the medium.

In summary, aBL demonstrates potential as a sustainable and low-cost decontamination technology for clinical environments and the food industry. Nevertheless, the theoretical dosimetric framework proposed in this study should be interpreted as a foundational benchmark rather than validated clinical protocols. Further in vivo investigations and clinical trials are mandatory to overcome the challenges of light attenuation in complex biological tissues and to ensure safety and efficacy in human applications. Beyond its effects on *E. coli*, the mechanistic nature of aBL offers a strategic perspective for the global efforts to mitigate antimicrobial resistance across various bacterial pathogens.

## Figures and Tables

**Table 1:** Main features of each study.

Author	Strain/Lineage	Wavelength	Technical Parameters	Bacterial Status	Main Result (Effectiveness)
[27]	EPEC CFT 073 and EAEC_042	400 nm	60 mW/cm^2^; 54–108 J/cm^2^	Planktonics/Biofilm	Reduction < 5-log; >90% reduction in biofilm seeding.
[28]	MDR Clones (ST10, ST131, ST648)	405 nm	155 mW/cm^2^; 139.5 J/cm^2^	Planktonicks	Reduction of 1.4 to 5.3 log_10_ (effective against multidrug-resistant bacteria).
[29]	*E. coli* ATCC 25922	460 nm	40 mW/cm^2^; 144 J/cm^2^	Planktonic/Meat	Reduction of 5.0 log (liquid) and 1.45 log (meat).
[30]	UPEC UTI89 and MG1655	405 nm	2.1–2.8 mW/cm^2^; 500 J/cm^2^	Planktonics/Biofilm	Reduction > 5-log in the log phase; resistance in the stationary phase.
[31]	*E. coli* (Clinical Strain)	405 nm	10.6–64.2 mW/cm^2^	Surface/Blood	Inactivation of approximately 7-log in agar; reduced efficacy in blood.
[32]	*E. coli* NCTC 9001	405 nm	10 to 90 mW/cm^2^	Planktonicks (Stress)	Synergy: accelerated inactivation under heat, salt, or acid.
[33]	*E. coli* ATCC 25922	405 nm	50 mW/cm^2^; 108 J/cm^2^	In vitro and In vivo	No resistance after 15 cycles; effective on wounds.
[34]	*E. coli* DH5α	425 nm	2.3 to 20.2 J/cm^2^	Planktonicks	Only 425 nm was effective; reduction with time/dose.
[35]	*E. coli* ATCC 25922	405 nm	120 mW/cm^2^; 540 J/cm^2^	Cow’s Milk	3-log reduction in skim milk; less in whole milk.
[36]	*E. coli* ATCC 25922 and Canine Isolates	465 nm	127 mW/cm^2^; 15–60 J/cm^2^	Planktonicks	Significant reduction (>3-log) from 45 J/cm^2^.
[37]	CPE *E. coli* (Superbugs: NDM, OXA-48)	405 nm	60 mW/cm^2^; 18–108 J/cm^2^	Biofilm (72 h)	Significant reduction in biofilm viability and seeding.

Note: All strains and lineages listed in this table refer to *Escherichia coli* species.

**Table 2:** Analysis of the Risk of Bias in studies.

Study	Design	Total Score	Quality (%)	OHAT Tier	Main Strength/Focus
[27]	In vitro	21/30	70.0%	Tier 2	Mature biofilms and clinical isolate diversity. +3
[28]	In vitro	22/30	73.3%	Tier 2	International MDR clones (KPC and MCR-1).
[29]	In vitro/Matrix	22/30	73.3%	Tier 2	Meat matrix and freshness (thermal control). +1
[30]	In vitro	22/30	73.3%	Tier 2	Synergistic effect of cold temperatures.
[31]	In vitro	22/30	73.3%	Tier 2	Light-diffusing fiber delivery system. +3
[32]	In vitro	22/30	73.3%	Tier 2	Synergy with acid and salt stress conditions.
[33]	In vitro/In vivo	23/30	76.6%	Tier 1/2	Explicit report of randomization in animal model. +2
[34]	In vitro	22/30	73.3%	Tier 2	Multi-spectral comparison (Red, Green, Blue). +3
[35]	In vitro/Matrix	22/30	73.3%	Tier 2	Complex matrix (milk) and fat content control.
[36]	In vitro	22/30	73.3%	Tier 2	Canine wound pathogens and active cooling system. +1
[37]	In vitro	22/30	73.3%	Tier 2	Carbapenemase-producing Enterobacteriaceae.

**Table 3:** Theoretical dosimetric framework for aBL photoinactivation of *E. coli* based on experimental evidence.

Biological Target/Application Scenario	Optimal Wavelength	Suggested Fluence (Dose)	Recommended Irradiance	Expected Outcome
[27] Planktonic (Log Phase)	405 nm	15–60 J/cm^2^	50–100 mW/cm^2^	>3-log reduction
[27] Mature Biofilms (72 h)	405 nm	100–200 J/cm^2^	>100 mW/cm^2^	Seeding inhibition (>90%)
[32] In Vivo (Wounds)	405–465 nm	45–100 J/cm^2^	Moderate (with active cooling)	Disinfection without cytotoxicity
[31] Complex Matrices (Meat/Milk)	405 nm	>150 J/cm^2^ (or Fractionated)	High (>120 mW/cm^2^)	Compensate for optical attenuation

**Table 4:** Potential clinical applications of aBL based on the *E. coli* pathotypes identified in the review.

*E. coli* Pathotype/Lineage	Associated Clinical Conditions	Potential aBL Application Scenario
[27] Uropathogenic (UPEC)	Urinary Tract Infections (UTIs), Cystitis	Intracavitary devices or catheter decontamination
[37] Enteroaggregative (EAEC)	Persistent Diarrhea, Gastroenteritis	Food surface decontamination/Water treatment
[33] MDR Clones (ST131, ST648)	Sepsis, Surgical Site Infections	Wound bed therapy/Pre-surgical skin antisepsis
[37] Commesal Laboratory Strains	Opportunistic Infections	Environmental surface disinfection

## Data Availability

The raw data supporting the conclusions of this article will be made available by the corresponding author on request.

## Abbreviations

The following abbreviations are used in this manuscript:

aBL	Antimicrobial Blue Light
ROS	Reactive Oxygen Species
CFU	Colony Forming Unit
MDR	Multidrug-Resistant
CPE	Carbapenemase-Producing *Enterobacteriaceae*
LED	Light-Emitting Diode
OHAT	Office of Health Assessment and Translation
PRISMA	Preferred Reporting Items for Systematic Reviews and Meta-Analyses
J/cm^2^	Joules per square centimeter (Fluence/Energy Dose)
mW/cm^2^	Milliwatts per square centimeter (Irradiance/Power Density)
